# Ten tips for teaching research integrity to early career students: A perspective over 20 years

**DOI:** 10.3389/frma.2022.989668

**Published:** 2022-08-26

**Authors:** Maruxa Martinez-Campos

**Affiliations:** ^1^Department of Medicine and Life Sciences, University Pompeu Fabra (MELIS-UPF), Barcelona, Spain; ^2^Barcelona Biomedical Research Park (PRBB), Barcelona, Spain

**Keywords:** research integrity, early career scientists, PhD students, training, research culture

## Abstract

Early Career Researchers (ECRs) are becoming increasingly aware of the importance of good scientific practices to ensure their work is trustworthy; but also of the effect that research culture has on those practices. Here I suggest ten tips on how best teach young researchers by incorporating their perspectives and needs. These are based on the lessons learned through our 20-year experience with a blended compulsory course for PhD students in a public university in Barcelona.

## Introduction

Scientific research is based on trust. Researchers trust work previously done by their colleagues, and society trusts the work of the scientific community (Ipsos MORI Report, 2017). The COVID-19 pandemic has highlighted more than ever the importance of this trust in science, especially in the health sciences field.

But there are fissures to this trust. Serious misconduct cases are not so common (Cyranoski, 2014), but there are many instances of unsound research, as seen with the general increase in retractions (Steen et al., 2013). So many that, in the last 20 years, they have led to talk of a “reproducibility crisis” (Ioannidis, 2005). Although fabrication, falsification and plagiarism are relatively rare (Fanelli, 2009), there are many questionable research practices (QRP), from using the wrong statistics to authorship conflicts (Flanagin et al., 1998; Martinson et al., 2005), which are much more frequent. Indeed, the recent National Survey on Research Integrity (NSRI) in the Netherlands found a prevalence of QRP over 50% (Gopalakrishna et al., 2022). Interestingly, researchers are more likely to spot such behaviors in others than in themselves (De Vries et al., 2006). There is still a third type of concerns adding to the lack of reliability of the scientific record; unintentional errors or honest mistakes, which account for 10–20% of retractions (Moylan and Kowalczuk, 2016). It is therefore clear that greater awareness about good scientific practices is essential in the research community. Indeed, training on Responsible Conduct of Research (RCR) is currently widespread, albeit very heterogeneous (Abdi et al., 2021).

The Biomedicine PhD programme from the Pompeu Fabra University (UPF) in Barcelona has been teaching a compulsory course on good research practices to first year students since 1998, when the Faculty was first established. The course, called “Science in Action”, was pioneer in Spain and it takes place twice a year, with about 60 students in each edition.

Both the content and the format of the course have evolved over the years as a result of the students' feedback (Table 1), making it more relevant and including their own interests and perceptions. Based on this experience, in this article I will share some tips on teaching research integrity by making the voices of Early Career Researchers heard.

**Table 1:** Sample of the comments and suggestions from “Science in action” students that have helped shape the current course, and on which the ten tips are based.

“I've been practicing science for many years, but this is the first time I've had formal discussion of many of the issues that arise”	Make it compulsory
“It made me think about a lot of very important things I didn't know existed!”	
“It has been surprisingly more interesting and stimulating than my expectations of a mandatory course, due to the tutors and students' positive participation”	
“I wish it should be compulsory for undergraduates, too”	
“Fruitful, entertaining and knowledgeable”	Make it fun
“Use of the role play is fun and helps thinking more”	
“The role-playing activities were my favorite ones”	
“I really liked the discussion formats. They were fun and interactive and we could share various ideas on things we may not have previously thought of”	
“I really enjoyed the seminar series, however, I wish there were more open-ended questions after each module”	Make it reflective
“It made me think a lot about the way I do my research; I am more aware now”	
“The seminar regarding the moral dilemmas was the most interesting to me”	
“It has been a great opportunity to share thoughts with my peers and to hear opinions and points of view that I had not considered before”	
“I enjoyed listening to people's differing opinions on a subject”	Make it safe
“I liked the format of being split into small groups and tackle different issues”	
“Do the seminars with less people so everyone can actively participate more”	
“I liked the idea of getting to know how my work colleagues think and realizing that many of us think the same. A highly recommended experience”	
“Sometimes the cases show the ‘supergood transparent choice'. Maybe we should discuss more things that are in the middle”	Make it relevant
“Particularly in terms of being reproducible and more rigorous, it is good to start these habits early in the PhD career, so this was especially relevant”	
“It would be great if we could discuss with 4th year PhD students or PIs to see different points of view, or real situations where research integrity was actually hard to follow”	
“Instead of talking about hypothetical situations, we could be doing case studies to see how these discussion pan out in real life”	
“The most useful part was the interactive sessions. Most of the situations in the theory are in a gray area, and this is very interesting to discuss latter in the live seminars”	Make it pro-active
“The interesting part was discussing with our colleagues real situations that can occur to any of us in the daily routine of science work”	
“Blended is perfect; you can have a look at the course before in your own time and come to the seminars with questions if needed”	Make it blended
“I like the fact that the live and online parts are not exactly the same, and that they complement each other”	
“Online courses give you the theory and with the seminars you can better understand it in an entertaining way”	
“It is good to have online material to make it work with my schedule”	
“It helped me re-think everything and feel empowered to change some things”	Make it systemic
“Living in my skin how a global change in the scientific community should be”	
“A realistic, but also encouraging, view of the research world that will make me a better researcher”	Make it positive
“It could be interesting to bring a PI to one of these courses or bring to them the conclusions of the students”	Make it a first step
“I wish this course could be repeated on the 3rd (and 2nd, 4th) year of PhD to discuss which problems we face then, has the first course helped, etc.”	

## Tips on teaching research integrity

### 1. Make it compulsory

Research integrity courses are essential during the early career of researchers, and they should be compulsory. Of course, voluntary courses have advantages—mainly that students will go with a better attitude and more interest. However, if done properly, even the skeptical students can enjoy the training and find it fruitful.

In our experience, first year PhD students tend to be busy and hesitant about the usefulness or value of courses like this one, and would be unlikely to choose such a course if given the choice. However, over the years, between 70 and 80% of them have rated the course as either excellent or very good.

In fact, according to the open anonymous comments received (Table 1), most students realize the course is very useful. A recurrent comment is that the seminars are a place where they feel they can discuss things they cannot discuss elsewhere—or they didn't even know about—and where they can find out about other researchers' opinions or experiences. In essence, it is a unique place to stop, reflect and share.

### 2. Make it fun

In order to engage students—especially the skeptics—it is important to make them protagonists and to include exercises or games that help create a relaxed and entertaining atmosphere. Research integrity is a serious matter, but it does not need to be boring.

Indeed, according to our surveys, most students end up being very happy with the course, among other things, because they have fun—something we believe is essential for them to be engaged and committed to the course (Chau, 2020).

For example, our last seminar consists of a role-play on authorship, where each student has to defend their position as an author of a paper, leading to very lively discussions. This is a fun exercise for the students and a good way to end the “Science in action” course with high spirits.

Another way of making discussions about integrity or ethics more gratifying is using films. These can be films created specifically to discuss such issues in the research world, such as interactive films like “The lab” from the US Office for Research Integrity (ORI), or “On being a scientist” from the University of Leiden. There are also many commercial movies that include ethical challenges and which can help bring some abstract ideas to life. A screening of such films followed by a guided debate can be an entertaining way of dealing with such ethical concepts.

### 3. Make it reflective

Although students are usually hoping for clear instructions on what to do, or detailed lists of Do's and Don'ts related to their day to day research practices, this should not be the aim of this course. This might be necessary, and could be done with further field-specific training. But on a first awareness raising course, what's important and most rewarding is that they discuss, share and reflect together about how to ensure a responsible conduct of research in the current scientific environment and share their own opinions and experiences with each other.

In this sense, we find that the use of the Dilemma game (Dilemma Game, Erasmus University Rotterdam) works well to make the students reflect about different situations, put themselves in other people's shoes and realize that things are not always black or white, but a shade of grays that depends on personality, the situation, the environment, etc.

We also encourage the students to come up with the topics to be discussed themselves, for example by asking them to identify which research culture aspects are currently challenging research integrity, and then reflecting together to find solutions.

### 4. Make it safe

In order to make these reflections most profitable it is important to create a safe environment, where the students feel they can share their views and experiences in an honest way without being judged. It is important to make it explicitly clear from the beginning that there are no wrong or right opinions and that they are all to be respected. Working in small groups also ensures everyone has a chance to speak and it is more comfortable and encouraging for those who are shy.

In our workshops, we try to have 20 people maximum, to ensure more fluidity and a friendly atmosphere where tricky issues can be openly talked about. Confidentiality and mutual respect are key, and this is made explicit at the beginning of the sessions. To further ensure an open and honest discussion, most of the activities are done in small groups of 4 or 5 people before having a wrap up discussion all together, so even those who are more reluctant have a chance to share their opinions.

### 5. Make it relevant

Whenever possible, use examples or case studies that the students can relate to, either from their own field of research or their position, and if possible use real examples. Indeed, a common critique among our students is that the examples and case studies normally used for discussion in these courses lack context or are not very realistic; the students would much rather hear about real cases they can relate to.

In that sense, another usual request is to involve senior researchers in the training. This request is two-fold. On the one hand, the students would like to hear about senior researcher's own experiences. But they also want the senior researchers to hear about what the students have to say. In our seminars the students reflect together on the motivations and frustrations of doing research and on what things of the current research culture they would like to change. After these issues have surfaced and they realize they all face the same challenges and concerns, the students feel more confident and see the benefit of their PIs or other senior researchers understanding their views, their worries and their expectations.

### 6. Make it pro-active

There are many methodologies you can use in the seminars (see Table 2 for an overview of some of the ones used in “Science in action”, with some pros and cons of each). But whichever you choose make sure they are student-centered techniques, where the students are encouraged to participate, listen, share and learn from each other before deciding what works best for them.

**Table 2:** Different methodologies used over the years for the “Science in action” course, and some of the advantages and challenges of each of them, in our experience.

Methodology	Advantages	Challenges
Case studies (real or customized), Dilemma cases	Clear and well defined, about different topics, you can use existing ones, can prepare them in advance.	Often not very realistic; even if detailed, they can lack many of the nuances and context information.
Oxford-style debate	Very good and entertaining way to discuss the pros and cons of a topic. It encourages students to find and defend arguments on both sides of any issue.	Only 4 people have a very active role, the rest are more passive. Works best for questions that have a yes or no answer (binary).
Role Plays	They can be fun, help guide the discussion. There are many existing ones to use, on different topics.	They depend a lot on the students, and how much they put themselves into the shoes of their character. Doesn't work so well when people are very shy.
Small group work—flipcharts and presentation of discussion	Ensures everyone participates, in the small group discussion and in the presentation.	Presentations can be repetitive if different groups are discussing the same topic. Some students might take a leading role and others be very passive, unless this is controlled.
World cafè	A good way of gathering collective knowledge from a big group of people, by making them discuss in small groups. It expands and enriches the conversation.	You need experienced facilitators to guide it, and to come up with good questions that are not too wide and not too specific. It can take quite a lot of time to do it properly.
Movies	They are entertaining and help put the problem/dilemma into context, make the students identify with the characters more easily than through written resources.	Apart from legal/copyright issues, the length might be a problem, and putting only a fragment might make it harder to understand all the complexities.

Over the years, we have used case studies, Oxford-style debates, role plays and other types of group work (Table 2). These kinds of active learning techniques not only ensure everyone's participation, but they also promote critical thinking and emotional engagement (McCarthy and Anderson, 2000), two aspects that ensure this research integrity training is relevant and appealing for all participants.

In fact, one of the more recurrent comments from the students is that they learned some specific tips, for example in record keeping, that their peers had shared during the seminars. This way they can take home some practical advice they can apply to their day-to-day work to make it more robust. Furthermore, this is an advice that is more likely to be incorporated because it comes from their own peers, who are in a similar situation to themselves and facing similar challenges.

### 7. Make it blended

Online content works well, giving busy students the freedom to learn at their own time and the flexibility to work around their lab experiments. But face-to-face seminars are fundamental to share views, to realize everyone is on the same boat, and learn from each other's experiences.

“Science in action” is a blended course. The first part, the theory modules and forums for discussion, is online and the students do it on their own time. There are also many extra resources—films, articles, other websites—for those who want to go deeper on a particular topic. The second part are the interactive seminars. These should be done in person, since, in our experience, online meetings remove much of the spontaneity and interactivity. In these seminars we try to put what they have learn into context in a holistic and practical way.

### 8. Make it systemic

Focus on the individual's accountability and their final choice and responsibility, but also discuss the role of the research environment at large, as well as the institution's own duties. In that sense, ensure that the institution has a code of conduct that all staff are aware of (in our case, the PRBB Good Scientific Practice Code, 2014). Regarding the content, although it might be worth tackling some topics on their own, it is also interesting to give a systemic view to see how everything is interconnected.

For example, issues not traditionally included in research integrity training, such as mental wellbeing (Levecque et al., 2017) or diversity and equality issues, are often discussed in our seminars because they are identified by the students themselves as playing an important role in the general research culture, in how research is done and in what type of researchers we want.

This is why, in the “Science in action” seminars we reflect together on the importance of personal ethics and the individual choice; but also on the scientific culture and its effects regarding research integrity in a holistic way. The students come up with what elements of the current research system make behaving with integrity difficult and then try to find solutions to these challenges from the point of view of different stakeholders: researchers, university or institutional management, funders and publishers.

### 9. Make it positive

It is easy to focus on the bad practices and their negative consequences, but it is more effective to highlight that research integrity can help them ensure their research is robust and trustworthy, helping them to be happier and more confident.

In “Science in action”, we discuss the effect of the environment on research practices, and invite students to come up with imaginative yet realistic solutions to current challenges. Importantly, we then offer real good practice examples by explaining some of the things already happening to encourage the students to investigate them further and to make them see they have a role to play, that they can effect change.

### 10. Make it a first step

Although very valuable, such an introductory course on research integrity is not enough on its own. It should be accompanied by further field-specific courses focused on techniques or tools, like experimental design and statistics, reproducibility, and other practical skills related to the robustness of research. But, most importantly, any training should be embedded in a general work environment that is safe, inclusive and open to discussions of concerns without fear of consequences. Ideally, the institutions where such courses are offered should have a Code of good conduct [for example, the PRBB Good Scientific Practice Code, 2014; ALLEA (All European Academies), 2017] or Research integrity policies (Bouter, 2020) and Research integrity promotion plans (Mejlgaard et al., 2020).

In that regard, the involvement of Principal Investigators and other senior researchers as role models is essential. Ways to achieve this are, for example, including research integrity training in introduction packages for all new employees or as a condition for being a PhD supervisor. Principal Investigators could also lead an annual informal research integrity discussion with their team, demonstrating their commitment to instilling a culture of integrity in their group. For the involvement of senior staff to be successful, it is important to ensure they realize that research integrity is not a barrier, but rather an essential ingredient to ensure the quality of their research, as well as to give them the necessary tools and guidance to lead the discussion.

## Take home messages

Independently of the format, duration or content of the training, there are three main key messages we try to get to the students:

- Although scientists are generally well intentioned, there is a “slippery slope between honest errors and intentional fraud” (Nylenna and Simonsen, 2006), and it is incredibly easy to ‘slip' through it and fool ourselves into getting the results we want (Nuzzo, 2015). Therefore, it is vital to be as careful as possible with our studies, our results and our assumptions—and to make sure we are our strongest critic and we remain skeptical of our own results.
- Things are not always black or white and often the way we choose to act is affected by the context or by pressures in the environment. It can no longer be denied that the current research culture and its perverse incentives have a great effect on the behavior of scientists (Gandevia, 2018; Global Business Ethics Survey Report, 2020), with some even calling it the “natural selection of bad science” (Smaldino and McElreath, 2016). According to a survey, for example, 62.8% of respondents admitted that the pressure to publish influences the way they report data (Boulbes et al., 2018). It is therefore important to reflect about the role of such research culture on research integrity and what we can do about it.
- No matter what the situation or the pressures involved, the final decision always belongs to the individual.

## Conclusion: Is teaching research integrity useful?

Some have raised concerns that courses on research integrity at this level may not be that effective in shaping the students' sense of integrity (Satalkar and Shaw, 2019). That may be so, and we cannot prove our course's effectiveness regarding a change of practice, neither. But the feedback received shows the course is arguably effective in achieving its declared aim: to raise awareness of research integrity among early career researchers, to make them reflect about issues that they normally would not think about, and talk to their peers about them. As one of the students concluded: “*Science in action* has provided me with theoretical and practical resources to improve my research. On the one hand with lots of tips to improve reproducibility and to properly communicate knowledge. On the other hand with the opportunity to share my experience with students in a similar position to mine”.

Although there will always be a minority of people who are dishonest no matter what, we believe courses on research integrity are beneficial for the vast majority of researchers, who despite being well-intentioned could easily find themselves behaving in less than optimal ways due to unawareness or unconscious biases. For those, such training might offer a unique opportunity to take into consideration issues that are sometimes ignored or given for granted.

Highlighting the importance of research integrity training in preventing reputational damage might also be a good way to get the senior management on board. As research culture plays a very important role in research integrity, it is essential that institutions lead the way forward. This can be done by providing training in the form of courses such as “Science in action”, but also by fostering informal discussions among the community (Thompson, 2019), establishing and promoting relevant policies and procedures or working toward a more responsible evaluation of researchers (Kretser et al., 2019). At the Barcelona Biomedical Research Park (PRBB), where the UPF MELIS department is hosted, our “Good scientific practice working group” has taken on the role of giving visibility to ethics and research integrity within our community of more than 1,300 people working at six different institutions, by organizing several activities. These have included a record keeping and data management campaign, or a World café on publications integrity with researchers at different career stages (see PRBB Good Scientific Practice Working Group Activities, 2014a,b).

In essence, I believe that improving research culture and driving culture change requires a coalition of forces at different levels: from formal training on research integrity with general courses like “Science in action” and other discipline-specific courses, to informal role modeling of senior scientists and discussions at the research group level, to activities and policies at the institutional level. Only the combination and involvement of all these levels will demonstrate that the institution takes research integrity seriously, that it cares.

## Data availability statement

The original contributions presented in the study are included in the article/supplementary material, further inquiries can be directed to the corresponding author.

## Author contributions

The author confirms being the sole contributor of this work and has approved it for publication.

## Conflict of interest

The author declares that the research was conducted in the absence of any commercial or financial relationships that could be construed as a potential conflict of interest.

## Publisher's note

All claims expressed in this article are solely those of the authors and do not necessarily represent those of their affiliated organizations, or those of the publisher, the editors and the reviewers. Any product that may be evaluated in this article, or claim that may be made by its manufacturer, is not guaranteed or endorsed by the publisher.
